# Differences in the mannose oligomer specificities of the closely related lectins from *Galanthus nivalis *and *Zea mays *strongly determine their eventual anti-HIV activity

**DOI:** 10.1186/1742-4690-8-10

**Published:** 2011-02-11

**Authors:** Bart Hoorelbeke, Els JM Van Damme, Pierre Rougé, Dominique Schols, Kristel Van Laethem, Elke Fouquaert, Jan Balzarini

**Affiliations:** 1 Rega Institute for Medical Research, K.U.Leuven, Minderbroedersstraat 10, B-3000 Leuven, Belgium; 2 Laboratory of Biochemistry and Glycobiology, Department of Molecular Biotechnology, Ghent University, Coupure Links 653, B-9000 Ghent, Belgium; 3 Signaux et Messages Cellulaires chez les Végétaux, UMR CNRS-UPS 5546, Pole de Biotechnologie végétale, BP 17, 24 Chemin de Borde Rouge, Castanet-Tolosan 31326, France

## Abstract

**Background:**

In a recent report, the carbohydrate-binding specificities of the plant lectins *Galanthus nivalis *(GNA) and the closely related lectin from *Zea mays *(GNA_maize_) were determined by glycan array analysis and indicated that GNA_maize _recognizes complex-type N-glycans whereas GNA has specificity towards high-mannose-type glycans. Both lectins are tetrameric proteins sharing 64% sequence similarity.

**Results:**

GNA_maize _appeared to be ~20- to 100-fold less inhibitory than GNA against HIV infection, syncytia formation between persistently HIV-1-infected HuT-78 cells and uninfected CD4^+ ^T-lymphocyte SupT1 cells, HIV-1 capture by DC-SIGN and subsequent transmission of DC-SIGN-captured virions to uninfected CD4^+ ^T-lymphocyte cells. In contrast to GNA, which preferentially selects for virus strains with deleted high-mannose-type glycans on gp120, prolonged exposure of HIV-1 to dose-escalating concentrations of GNA_maize _selected for mutant virus strains in which one complex-type glycan of gp120 was deleted. Surface Plasmon Resonance (SPR) analysis revealed that GNA and GNA_maize _interact with HIV III_B _gp120 with affinity constants (K_D_) of 0.33 nM and 34 nM, respectively. Whereas immobilized GNA specifically binds mannose oligomers, GNA_maize _selectively binds complex-type GlcNAcβ1,2Man oligomers. Also, epitope mapping experiments revealed that GNA and the mannose-specific mAb 2G12 can independently bind from GNA_maize _to gp120, whereas GNA_maize _cannot efficiently bind to gp120 that contained prebound PHA-E (GlcNAcβ1,2man specific) or SNA (NeuAcα2,6X specific).

**Conclusion:**

The markedly reduced anti-HIV activity of GNA_maize _compared to GNA can be explained by the profound shift in glycan recognition and the disappearance of carbohydrate-binding sites in GNA_maize _that have high affinity for mannose oligomers. These findings underscore the need for mannose oligomer recognition of therapeutics to be endowed with anti-HIV activity and that mannose, but not complex-type glycan binding of chemotherapeutics to gp120, may result in a pronounced neutralizing activity against the virus.

## Background

Lectins represent a heterogeneous group of carbohydrate-binding proteins that are present in different species (e.g. prokaryotes, plants, invertebrates and vertebrates) and vary in size, structure and ability (affinity for different glycan determinants) to bind carbohydrates. Plant lectins represent a large group of proteins classified into twelve families, each typified by a particular carbohydrate binding motif [1]. At present, most studies have dealt with plant lectins classified as legume lectins, chitin-binding lectins, type 2 ribosome inactivating proteins and monocot mannose-binding lectins (MMBLs). After the identification of the first reported MMBL from snowdrop bulbs, namely *Galanthus nivalis *agglutinin (GNA) [2], lectins were isolated and characterized from other closely related plant species. Similar lectins were also identified outside plants, for example in the fish *Fugu rubripes *[3] and in several Pseudomonas spp. [4,5]. GNA is the prototype of a family of lectins that resemble each other with respect to their amino acid sequences, three-dimensional structures, and sugar-binding specificities. The lectin subunits of this class contain similar structural features, containing a β-barrel composed of 3 antiparallel four-stranded β sheets [6].

Members of the GNA-related lectins have been investigated for their antiviral activity (in particular HIV). Indeed, the plant lectins *Galanthus nivalis *agglutinin (GNA) and *Hippeastrum *hybrid agglutinin (HHA) have been described to inhibit viral entry [7,8], presumably by their interaction with the glycans on HIV gp120. It has been reported that these carbohydrate binding agents (CBAs) block virus entry by inhibiting the fusion of cell-free HIV particles with their target cells. Also, they prevent the capture of virions by the DC-SIGN-receptor present on dendritic cells of the innate immune system and efficiently inhibit the subsequent transmission of the virus to CD4^+ ^T-cells. Besides blocking HIV entry, CBAs have also the ability to select for virus strains in which one or more glycans on gp120 are deleted. This mechanism of drug escape results in the exposure of previously hidden immunogenic epitopes on the virus envelope glycoproteins [9].

Until recently, most plant lectin research was limited to vacuolar plant lectins which have the advantage of being present at relatively high quantities in seeds. Nowadays, nucleocytoplasmic plant lectins can also be efficiently isolated, even though they occur at low concentrations in the plant tissues. One example of a nucleocytoplasmic plant lectin is the maize homolog of the vacuolar GNA [10]. This GNA-like lectin from *Zea mays *(GNA_maize_) of which the gene was cloned and expressed in *Pichia pastoris *by Fouquaert and co-workers [10] shows 64% sequence similarity with GNA from snowdrop.

All the reported GNA-related lectins including GNA_maize _have homologous sequences and structural similarities. Despite this similarity at the protein level, this class of lectins may display important differences in the post-translational processing of the precursors [6]. Many GNA-related lectins are indeed synthesized as prepro-proteins and then converted in the mature polypeptide by the co-translational cleavage of a signal peptide and the post-translational removal of a C-terminal peptide [10]. However, more recently it was shown that some GNA-related lectins are synthesized without a signal peptide and as a consequence are located in the nucleocytoplasmic compartment of the plant cell. This processing results in a different subcellular localization of the lectin. The GNA homolog from maize (GNA_maize_) is processed in such a way and is, therefore, in contrast to the vacuolar GNA, located in the cytoplasm [10,11].

Native GNA is a tetrameric protein of 50 kDa with three carbohydrate-binding motifs in each monomer and was originally isolated from snowdrop bulbs [2]. GNA was originally described as a lectin with a specificity towards Manα1,3Man-containing oligosaccharides [12]. The molecular mass of the native recombinant GNA_maize _is 60 kDa and the lectin exists also as a tetramer with 3 carbohydrate-binding sites per monomer [11]. However, it was reported before that gene divergence may have a serious impact on the carbohydrate-binding potential of lectins [13]. Sequence alignments revealed that only the third carbohydrate-binding site (CBS) is similar between the GNA_maize _and the GNA lectin, whereas the first and second CBS differ with only 2 and 1 amino acid changes, respectively [11]. However, glycan microarray analysis revealed striking differences in glycan specificity. GNA_maize _interacts preferentially with complex-type glycans, whereas GNA almost exclusively binds to high-mannose-type glycans [11]. Fouquaert and colleagues hypothesized that this difference in glycan-binding properties reflects the ~100-fold decreased anti-HIV-1 activity of GNA_maize _when compared to GNA [11].

To reveal in more detail the correlation between gene divergency of GNA and GNA_maize_, as well as the change in carbohydrate-binding specificity and differences in anti-HIV activity, we now report a detailed study of GNA_maize _(in comparison with GNA) covering its anti-HIV activity, its kinetic interaction with the HIV-1 envelope glycoprotein gp120, epitope mapping experiments to determine its glycan specificity on gp120 and its antiviral resistance spectrum.

## Methods

### Test compounds

The mannose-specific plant lectin GNA from snowdrop and the cytoplasmatic GNA_maize _from maize were derived and purified as described previously [2,11]. GlcNAcß1,2Man, (α1,3-man)_2 _and (β1,4-GlcNAc)_3 _were obtained from Dextra Laboratories (Reading, UK). (α1,2-man)_3 _was purchased from Carbohydrate Synthesis (Oxford, UK). The anti-gp120 2G12 mAb was obtained from Polymun Scientific GmbH (Vienna, Austria). The lectins *Phaseolus vulgaris *Erythroagglutinin (PHA-E) and *Sambucus nigra *agglutinin (SNA) from elderberry were from Vector Laboratories (Peterborough, UK).

### Cells

Human T-lymphocytic CEM, C8166, HuT-78 and Sup-T1 cells were obtained from the American Type Culture Collection (Manassas, VA, USA). The Raji/DC-SIGN cells were constructed by Geijtenbeek *et al*. [14] and kindly provided by L. Burleigh (Institut Pasteur, Paris, France). Persistently HIV-infected HuT-78/HIV cells were obtained upon cultivation for 3 to 4 weeks of HuT-78 cell cultures exposed to HIV-1(III_B_). All cell lines were cultivated in RPMI-1640 medium (Invitrogen, Merelbeke, Belgium) supplemented with 10% fetal bovine serum (FBS) (BioWittaker Europe, Verviers, Belgium), 2 mM L-glutamine, 75 mM NaHCO_3 _and 20 μg/ml gentamicin (Invitrogen).

### Viruses

HIV-1(III_B_) and HIV-1(BaL) were a kind gift from R.C. Gallo (Institute of Human Virology, University of Maryland, Baltimore, MD) (at that time at the NIH, Bethesda, MD) and HIV-2(ROD) was provided by L. Montagnier (at that time at the Pasteur Institute, Paris, France). The following clinical isolates were used: UG273 (clade A, R5), DJ259 (clade C, R5) and ID12 (clade A/E, R5).

### Antiretrovirus assays

CEM cells (5 × 10^5 ^cells per ml) were suspended in fresh culture medium and infected with HIV-1 and HIV-2 at 100 times the CCID_50 _(50% cell culture infective doses) per ml of cell suspension, of which 100 μl was mixed with 100 μl of the appropriate dilutions of the test compounds, and further incubated at 37°C. After 4 to 5 days, syncytia formation was recorded microscopically in the cell cultures. The 50% effective concentration (EC_50_) corresponds to the compound concentration required to prevent syncytium formation by 50% in the virus-infected CEM cell cultures.

Buffy coat preparations from healthy donors were obtained from the Blood Bank in Leuven. Peripheral blood mononuclear cells (PBMC) were isolated by density gradient centrifugation over Lymphoprep (density = 1.077 g/ml; Nycomed, Oslo, Norway). The PBMC were transferred to RPMI 1640 medium supplemented with 10% fetal calf serum (BioWhittaker Europe) and 2 mM L-glutamine and then stimulated for 3 days with phytohemagglutinin (PHA; Murex Biotech Limited, Dartford, United Kingdom) at 2 μg/ml. HIV-infected or mock-infected PHA-stimulated blasts were cultured in the presence of 10 ng of interleukin-2/ml and various concentrations of GNA and GNA_maize_. Supernatant was collected at days 8 to 10, and HIV-1 core antigen in the culture supernatant was analyzed by the p24 core antigen enzyme-linked immunosorbent assay (ELISA; DuPont-Merck Pharmaceutical Co., Wilmington, Del.).

### Co-cultivation assay between Sup-T1 and persistently HIV-1-infected HuT-78 cells

Persistently HIV-1(III_B_)-infected HuT-78 cells (designated HuT-78/HIV-1) were washed to remove cell-free virus from the culture medium, and 5 × 10^4 ^cells (50 μl) were transferred to 96-well microtiter plates. Next, a similar amount of Sup-T1 cells (50 μl) and appropriate concentrations of test compound (100 μl), were added to each well. After 1 to 2 days of co-culturing at 37°C, the EC_50 _values were quantified based on the appearance of giant cells by microscopical inspection.

### Capture of HIV-1(III_B_) by Raji/DC-SIGN cells and subsequent co-cultivation with C8166 cells

The experiment was performed as described previously [15]. Briefly, B-lymphocyte DC-SIGN-expressing (Raji/DC-SIGN) cells were suspended in cell culture medium at 2 × 10^6 ^cells/ml. 100 μl of HIV-1(III_B_) (~250,000 pg p24) were added in the presence of 400 μl of serial dilutions of the test compounds. After 60 minutes of incubation, the cells were carefully washed 3 times to remove unbound virions and resuspended in 1 ml of cell culture medium. The captured HIV-1(III_B_) was quantified by a p24 Ag ELISA. From the Raji/DC-SIGN cell suspension, 200 μl were also added to the wells of a 48-well microtiter plate in the presence of 800 μl uninfected C8166 cells (2.5 × 10^5 ^cells/ml). These cocultures were further incubated at 37°C, and syncytia formation was evaluated microscopically after ~ 18 to 42 h, and viral p24 Ag determination in the culture supernatants was performed.

### Selection and isolation of GNA_maize_-resistant HIV-1 strains

CEM cells were infected with HIV-1(III_B_) and seeded in 48-well plates in the presence of GNA_maize _at a concentration equal to one- to two-fold its EC_50_. Three independent series of subcultivations were performed for GNA_maize_. The compound concentration was increased stepwise (~ 1.5-fold) when full cytopathic effect was detected. Subcultivations occurred after every 4 to 5 days by transferring 100 μl cell suspension of the GNA_maize_-exposed HIV-infected cells to 900 μl uninfected CEM cell cultures.

### Genotyping of the HIV-1 env region

Viral RNA was extracted from virus supernatants using the QIAamp Viral RNA Mini Kit (Westburg, Heusden, the Netherlands). The genotyping of both *Env *genes, gp120 and gp41, were determined in this assay as described previously [16].

### Surface plasmon resonance (SPR) analysis

Recombinant gp120 proteins from HIV-1(III_B_) (ImmunoDiagnostics Inc., Woburn, MA), one batch produced by CHO cell cultures and another by insect cells (Baculovirus) were covalently immobilized on a CM5 sensor chip in 10 mM sodium acetate, pH 4.0, using standard amine coupling chemistry. The exact chip densities are summarised in the results section. A reference flow cell was used as a control for non-specific binding and refractive index changes. All interaction studies were performed at 25°C on a Biacore T100 instrument (GE Healthcare, Uppsala, Sweden). The plant lectins GNA and GNA_maize _were serially diluted in HBS-P (10 mM HEPES, 150 mM NaCl and 0.05% surfactant P20; pH 7.4) supplemented with 0.2 mM Ca^2+^, covering a wide concentration range by using two-fold dilution steps. Samples (often in duplicate) were injected for 2 minutes at a flow rate of 45 μl/min and the dissociation was followed for 8 minutes. Several buffer blanks were used for double referencing. The CM5 sensor chip surface was regenerated with 1 injection of 50 mM NaOH and with 1 injection of Glycine-HCl pH 1.5 for GNA_maize _and GNA, respectively. All studied interactions resulted in specific binding signals. The shape of the association and dissociation phases reveals that the curves are not following 1:1 Langmuir kinetics. The experimental data were fit using the 1:1 binding model (Biacore T100 Evaluation software 2.0.2) to determine the binding kinetics. These affinity and kinetic values are apparent values as the injected concentrations of the evaluated compounds did result in biphasic binding signals.

To generate more information on the glycan specificity of GNA_maize _and GNA, three different SPR-based experiments were performed. In the first set-up, the sensor chip was immobilized with GNA and GNA_maize _and binding with the (α1,2-man)_3_, (α1,3-man)_2_, (β1,4-GlcNAc)_3_, and GlcNAcß1,2Man analytes was examined as described above. The experimental data were fit using the steady-state affinity model (Biacore T100 Evaluation software 2.0.2) to determine the apparent K_D_-values. In the second set-up, a competition assay of GNA_maize_, GNA and the anti-gp120 2G12 mAb for binding to immobilized HIV-1 gp120 was performed in which one of each of the compounds was administered for 2 minutes to immobilized gp120 and by the end of this time period, the initial compound concentration was sustained but now in the additional presence of one of the two other compounds. In a third set-up, a competition experiment for binding of GNA, GNA_maize _and the mAb 2G12 to HIV-1 gp120 was performed with PHA-E (prefers binding to GlcNAcß1,2man- and Galß1,4GlcNAc determinants) and SNA (prefers binding to NeuAcα2,6- and to a lesser degree NeuAcα2,3-X determinants).

### Molecular modeling

Homology modeling of GNA_maize _was performed on a Silicon Graphics O2 10000 workstation, using the programs InsightII, Homology and Discover (Accelrys, San Diego CA, USA). The atomic coordinates of GNA complexed to mannose (code 1MSA) [17] were taken from the RCSB Protein Data Bank [18] and used to build the three-dimensional model of the GNA-like lectin from maize. The amino acid sequence alignment was performed with CLUSTAL-X [19] and the Hydrophobic Cluster Analysis (HCA) [20] plot was generated http://mobyle.rpbs.univ-paris-diderot.fr/cgi-bin/portal.py?form=HCA to recognize the structurally conserved regions common to GNA and GNA_maize_. Steric conflicts resulting from the replacement or the insertion of some residues in the modeled lectin were corrected during the model building procedure using the rotamer library [21] and the search algorithm implemented in the Homology program [22] to maintain proper side-chain orientation. Energy minimization and relaxation of the loop regions were carried out by several cycles of steepest descent using Discover3. After correction of the geometry of the loops using the minimize option of TurboFrodo, a final energy minimization step was performed by 100 cycles of steepest descent using Discover 3, keeping the amino acid residues forming the carbohydrate-binding sites constrained. The program TurboFrodo (Bio-Graphics, Marseille, France) was used to draw the Ramachandran plots [23] and perform the superimposition of the models. PROCHECK [24] was used to check the stereochemical quality of the three-dimensional model: 74.8% of the residues were assigned to the most favourable regions of the Ramachandran plot (77.6% for GNA). Cartoons were drawn with Chimera [25].

Molecular surface and electrostatic potentials were calculated and displayed with GRASP using the parse3 parameters [26]. The solvent probe radius used for molecular surfaces was 1.4 Å and a standard 2.0 Å-Stern layer was used to exclude ions from the molecular surface [27]. The inner and outer dielectric constants applied to the protein and the solvent were fixed at 4.0 and 80.0, respectively, and calculations were performed keeping a salt concentration of 0.145 M. Surface topology of the carbohydrate-binding sites was rendered and analyzed with PyMol (W.L. DeLano, http://pymol.org).

The docking of methyl mannose (MeMan) into the carbohydrate-binding sites of GNA_maize _was performed with the program InsightII (Accelrys, San Diego CA, USA). The lowest apparent binding energy (E_bind _expressed in kcal.mol^-1^) compatible with the hydrogen bonds (considering Van de Waals interactions and strong [2.5 Å < dist(D-A) < 3.1 Å and 120° < ang(D-H-A)] and weak [2.5 Å < dist(D-A) < 3.5 Å and 105° < ang(D-H-A) < 120°] hydrogen bonds; with D: donor, A: acceptor and H: hydrogen) found in the GNA/Man complex (RCSB PDB code 1MSA) [17] was calculated using the forcefield of Discover3 and used to anchor the pyranose ring of the sugars into the binding sites of the lectin. The positions of mannose observed in the GNA/Man complex were used as starting positions to anchor mannose in the carbohydrate-binding sites of GNA_maize_. Cartoons showing the docking of MeMan in the mannose-binding sites of the lectins were drawn with Chimera and PyMol.

## Results

### Antiviral activity of GNA and GNA_maize _against HIV-1(III_B_) and HIV-2(ROD) infection

**Figure 1:**
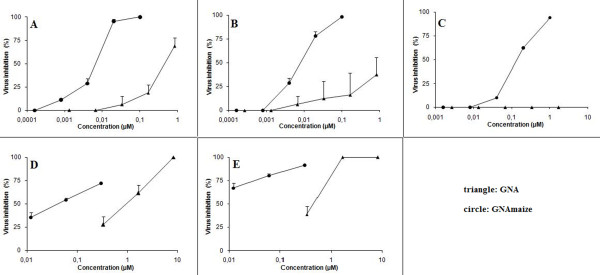
**Antiviral activity of GNA (black triangle) and GNA_maize _(black circle) in cell culture**. Inhibitory activity against HIV-1(III_B_) (Panel A) and HIV-2(ROD) (Panel B), respectively, in CEM cell cultures. Panel C: Inhibitory activity against HIV-1(III_B_) in cocultivation of HuT78/HIV-1 with SupT1. Panels D and E: Inhibitory activity against DC-SIGN-mediated capture of HIV-1(III_B_) by Raji/DC-SIGN (Panel D) and subsequent virus transmission to CD4^+ ^T-cells (Panel E).

GNA and GNA_maize _inhibited the HIV-1- and HIV-2-induced cytopathic effect in CEM cell cultures (Table 1 and Figure 1, Panels A and B). The EC_50 _(50% effective concentration) values of GNA for HIV-1(III_B_) and HIV-2(ROD) were 0.007 μM and 0.008 μM, respectively. GNA_maize _was found to be much less active against the two virus strains with EC_50_-values of 0.46 μM and >0.83 μM, respectively. Thus, GNA is ~60 to ≥100-fold more potent as an anti-HIV agent than GNA_maize_. A similar phenomenon is also observed for their activity against several HIV-1 clade clinical isolates tested in PBMC (Table 2).

**Table 1:** Anti-HIV activity of GNA_maize _and GNA in different cell systems

CBA	**HIV-1(III**_**B**_**) EC**_**50**_^**a **^**(μM)**	**HIV-2(ROD) EC**_**50**_^**a **^**(μM)**	**HuT-78/HIV-1 + Sup T1 EC**_**50**_^**b **^**(μM)**
GNA_maize_	0.46 ± 0.13	≥ 0.83	>1.67

GNA	0.007 ± 0.001	0.008 ± 0.001	0.062 ± 0.064

^a^50% Effective concentration or compound concentration required to inhibit virus-induced cytopathicity in CEM cell cultures by 50%.

^b^50% Effective concentration or compound concentration required to inhibit syncytia formation between HuT-78/HIV-1 and Sup-T1 cells by 50%.

Data are means of at least two to four independent experiments.

**Table 2:** Antiviral activity of GNA_maize _and GNA in PBMC against clinical isolates

CBA	**EC**_**50**_^**a **^**(μM)**			
	
	Clade A, UG273	Clade B, BaL	Clade C, DJ259	Clade A/E, ID12
GNA_maize_	1.4	>1.6	>1.6	>1.6
GNA	0.046	0.13	0.84	0.38

^a^50% Effective concentration or compound concentration required to inhibit p24 production of HIV-infected PBMC.

### Activity of CBAs on syncytia formation in co-cultures between HuT-78/HIV-1 and Sup-T1 cells

GNA_maize _could not efficiently prevent syncytia formation between persistently HIV-1(III_B_)-infected HuT-78/HIV-1 cells and uninfected CD4^+ ^T-lymphocyte SupT1 cells (EC_50 _>1.7 μM), whereas GNA was able to prevent syncytia formation in the co-cultures at an EC_50 _of 0.062 μM (Table 1 and Figure 1, Panel C).

### Effect of GNA and GNA_maize _on the capture of HIV-1 by Raji/DC-SIGN cells and on subsequent virus transmission to uninfected CD4^+ ^T-cells

We also investigated the potential of GNA_maize _to prevent HIV-1(III_B_) capture by DC-SIGN using Raji cells transfected with DC-SIGN; and, next, the potential to decrease the transmission of DC-SIGN-captured virions to uninfected CD4^+ ^T-lymphocyte C8166 cells. HIV-1 was shortly (30 minutes) exposed to different GNA and GNA_maize _concentrations before the virus was added to the DC-SIGN-expressing Raji/DC-SIGN cells. One hour later, free virus particles and the test compounds were carefully removed from the cell cultures by several washing steps. P24 Ag ELISA analysis revealed that GNA_maize _dose-dependently inhibited HIV-1(III_B_) capture by Raji/DC-SIGN cells with an EC_50 _of 0.90 μM. In this assay, GNA was 20-fold more potent in inhibiting virus capture than GNA_maize _(Table 3 and Figure 1, Panel D). Next, the washed GNA_maize_/GNA-treated HIV-1-exposed Raji/DC-SIGN cells were co-cultured with CD4^+ ^T-lymphocytes C8166 cells and syncytia formation was recorded microscopically within 24 to 48 hours after co-cultivation. GNA_maize _inhibited HIV-1 transmission at an EC_50 _of 0.44 μM which was 70-fold less efficient than GNA (Table 3 and Figure 1, Panel E).

**Table 3:** Inhibitory activity of GNA_maize _and GNA on DC-SIGN-mediated capture of HIV-1(III_B_) by DC-SIGN^+ ^cells and subsequent virus transmission to CD4^+ ^T cells

CBA	**EC**_**50**_^**a **^**(μM)**	
	
	Capture	Transmission
GNA_maize_	0.90 ± 0.40	0.44 ± 0.09
GNA	0.04 ± 0.01	0.006 ± 0.005

^a^50% Effective concentration required to inhibit HIV-1 capture by DC-SIGN and subsequent transmission to CD_4_^+ ^T-cells.

### Selection of GNA_maize _-resistant HIV-1(III_B_) strains and determination of mutations in the gp160 gene of GNA_maize_-exposed HIV-1(III_B_) strains

**Figure 2:**
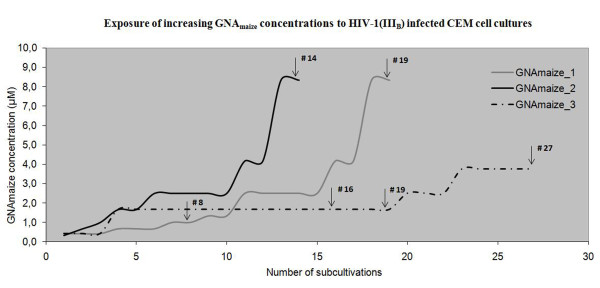
**Selection of GNA_maize _resistance development in HIV-1(III_B_)-infected CEM cell cultures**. Arrows indicate the time points where virus isolates were taken for further characterisation. GNA_maize__1, GNA_maize__2 and GNA_maize__3 represent three independent subcultivation schedules.

HIV-1(III_B_)-infected CEM cell cultures were exposed to a GNA_maize _concentration comparable to its EC_50_. Three independent series of GNA_maize _selections were done (Figure 2). Subcultivations were performed every 4 to 5 days. Virus-induced giant cell formation was recorded microscopically, and the drug concentration was increased 1.5-fold when full cytopathic effect was scored. Virus isolates were taken (arrows in Figure 2) during the selection process and analyzed for amino acid changes in the viral envelope gene (encoding for gp120 and gp41). Two different mutations were observed in putative N-glycosylation motifs in gp120 and one mutation in gp41 when considering all virus isolates that were subjected to genotypic analysis (Table 4). The virus isolates at passages GNA_maize__1#8, GNA_maize__1#19, GNA_maize__2#14, GNA_maize__3#19 and GNA_maize__3#27 contained only one N-glycosylation site deletion in gp120, being N/Y301Y. The deleted N-glycan in gp120 found to occur in the GNA_maize _selection experiments (N301) was previously determined as a complex-type glycan [28]. One new N-glycosylation motif appeared at amino acid position 29 in gp120 of virus isolate GNA_maize__3#16. In this virus isolate a single N-glycosylation site deletion in gp41 was observed at amino acid position 811NAT/I813.

**Table 4:** Amino acid mutations that appeared in the envelope of HIV-1(III_B_) strains under sustained GNA_maiz__e _or GNA pressure

**putative glycosylation motifs in HIV-1(III**_**B**_**) gp160**	type of N-glycan	**GNA**_**maize**_**_1#8**	**GNA**_**maize**_**_1#19**	**GNA**_**maize**_**_2#14**	**GNA**_**maize**_**_3#16**	**GNA**_**maize**_**_3#19**	**GNA**_**maize**_**_3#27**	**GNA**^**c**^
					*S29[N,S]^b^*			
								
							A48T	
				K59[K,E]				
							A70T	
88NVT90	complex							**T90[T/I]**
			V101[I,V]			V101[I,V]		
		H105[N,H]						
136NDT138	complex							
141NSS143	complex							
156NCS158	complex							
160NIS162	complex							
							F175L	
186NDT188	complex							
								
197NTS199	complex							
230NKT232	high mannose							**T232M**
234NGT236	high mannose							**N234K**
241NVS243	high mannose							
262NGS264	high mannose							
							E268K	
276NFT278	complex							
289NQS291	high mannose							**N289[N,D]**
								**S291[S,F]**
								
295NCT297	high mannose							
301NNT303	complex	**[N,Y]301Y**	**[N,Y]301Y**	**[N,Y]301Y**		**[N,Y]301Y**	**[N,Y]301Y**	**[N,Y]301Y**
								
					A329[T,A]			
332NIS334	high mannose							
339NNT341	high mannose							**T341I**
								
356NKT358	complex							
					G379[E,G]			
386NST388	high mannose							
								
392NST394	high mannose							**T394I**
397NST399	complex							
401NNT403	complex							
							G404R	
				G410[E,G]				
			A433[T,A]			A433[T,A]		
					A436[T,A]			
448NIT450	high mannose							
		G458[S,G]						
								
463NGS465	complex							
				G471[E,G]				

606NAS608	N.D.^a^							
611NKS613	N.D.							
620NMT622	N.D.							
632NYT634	N.D.							
669NIT671	N.D.							
745NGS747	N.D.							
811NAT813	N.D.			**T813[T,I]**				

^a^No assignment of the nature of the glycans was found back in the literature.

^b^This amino acid change results in the creation of a new putative *N*-glycosylation site (italics).

Assignment of high mannose- or complex type glycans according to Leonard *et al*. [28]. Amino acid sequence numbering according to Kwong *et al*. [47].

Mutated amino acids in bold result in the deletion of a glycosylation motif.

^c ^Data taken from Balzarini *et al*. [35].

^d ^This glycosylation motif is present in HIV-1(NL4.3), but not in HIV-1(III_B_).

### Kinetic analysis of the interaction of GNA and GNA_maize _with HIV-1 III_B _gp120

**Figure 3:**
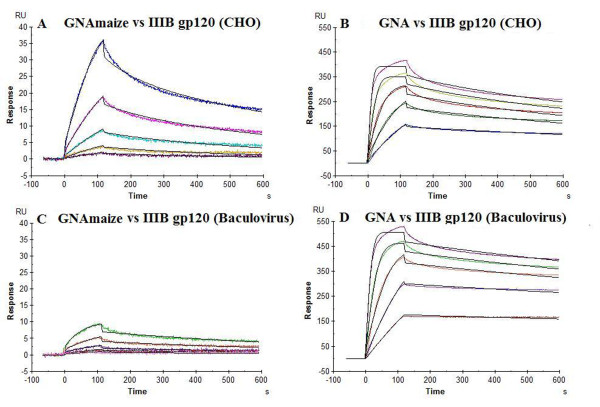
**Kinetic analysis of the interactions of GNA_maize _(A, C) and GNA (B, D) with immobilized HIV-1 III_B _gp120 isolated from CHO cell cultures and from Baculovirus using SPR technology**. Serial two-fold analyte dilutions (covering a concentration range from 5 to 80 nM and from 39 to 625 nM, respectively) were injected over the surface of the immobilized gp120. The experimental data (coloured curves) were fit using the 1:1 binding model (black lines) to determine the kinetic parameters. The data are a representative example of three independent experiments. The biosensor chip density was 822 RU for gp120 from CHO (or 6.9 fmol gp120) (panels A & B) and 725 RU for gp120 from Baculovirus (or 6.0 fmol gp120) (panels C & D).

The interaction of both plant lectins with HIV-1 gp120 was subjected to a detailed kinetic characterization by surface plasmon resonance (SPR) analysis. GNA_maize _and GNA were evaluated against HIV-1(III_B_) gp120, derived from either mammalian CHO cells and from insect cells (Baculovirus system). Two-fold serial dilution series of GNA and GNA_maize _(covering a concentration range of 5 to 80 nM and 39 to 625 nM, respectively) were applied to the gp120 immobilized on a CM5 sensor chip. A 1:1 Langmuir kinetic fit was applied to obtain the apparent kinetic association rate constant k_a _(k_on_, on-rate) and dissociation rate constant k_d _(k_off_, off-rate) and the apparent affinity constant K_D _(ratio k_d_/k_a_) (Table 5; Figure 3). A ~100-fold difference in K_D_-value was detected between both plant lectins when evaluated against HIV-1 gp120 (CHO cell-derived). The apparent affinity of GNA for gp120 was K_D _= 0.33 nM, whereas that of GNA_maize _was K_D _= 34 nM. The k_on_-values differed by a factor of ~ 20 and the k_off_-values by ~ 5-fold. GNA has a two-fold better affinity and GNA_maize _a 2-fold weaker affinity for HIV-1 gp120 (insect cell-derived) compared to HIV-1 gp120 (CHO cell-derived).

**Table 5:** Kinetic data for the interaction of GNA and GNA_maize _with immobilized HIV-1 III_B _gp120

	**K**_**D **_**(nM)**	**k**_**a **_**(1/Ms)**	**k**_**d **_**(1/s)**
**GNA *vs *III_B _gp120 (CHO)**	0.33 ± 0.07	(2.81 ± 0.68) E+06	(9.00 ± 1.14) E-04
**GNA *vs *III_B _gp120 (Baculovirus)**	0.17 ± 0.12	(2.75 ± 1.56) E+06	(3.63 ± 0.75) E-04
**GNA_maize _*vs *III_B _gp120 (CHO)**	34 ± 13	(1.37 ± 0.78) E+05	(5.24 ± 4.50) E-03
**GNA_maize _*vs *III_B _gp120 (Baculovirus)**	77 ± 17	(2.23 ± 0.74) E+04	(1.64 ± 0.20) E-03

### Affinity analysis for the interactions of various oligosaccharides with GNA_maize _and GNA

**Figure 4:**
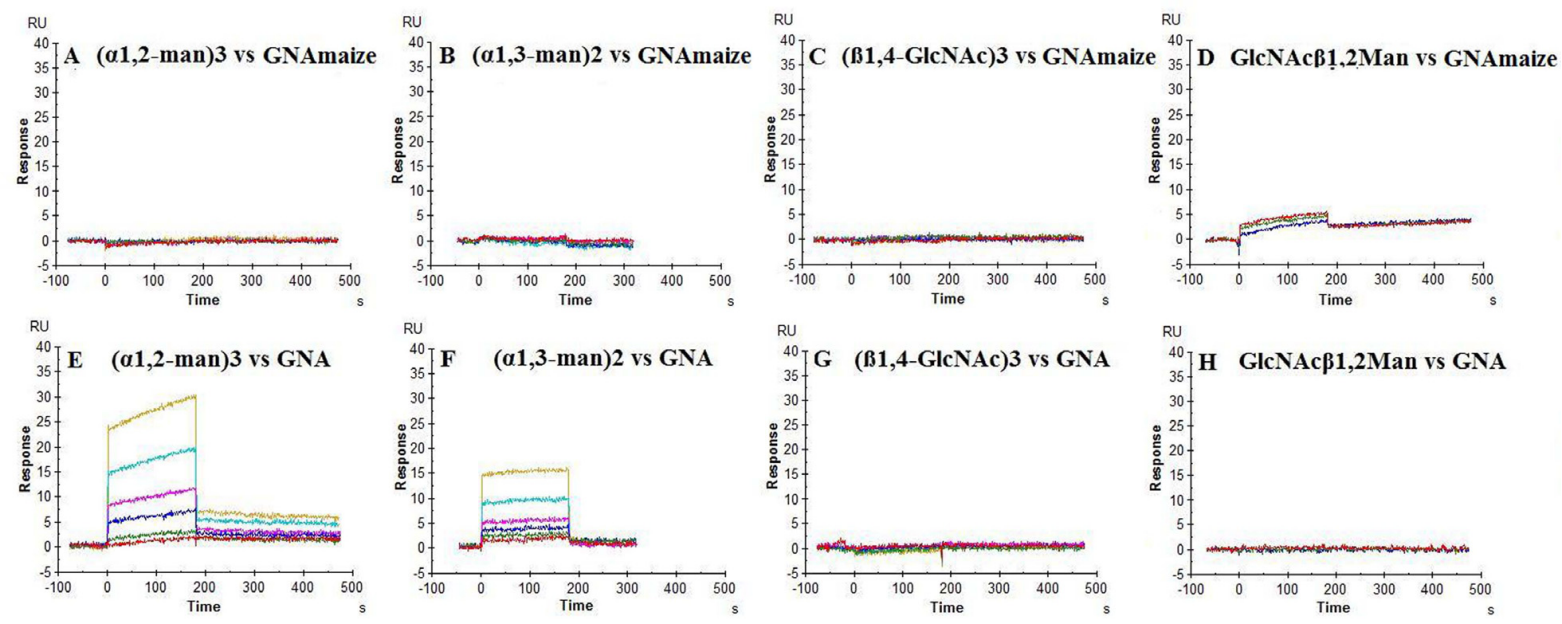
**Affinity analysis of (α1,2-man)_3_, (α1,3-man)_2_, (β1,4-GlcNAc)_3 _and GlcNAcß1,2Man with immobilized GNA_maize _and GNA**. Serial two-fold analyte dilutions were injected over the surface of the GNA_maize_- (Panels A to D)- or GNA- (Panels E to H)-bound sensor chip. These dilutions covered a concentration range from 7.8 to 1000 μM for (α1,2-man)_3_, and (β1,4-GlcNAc)_3 _(Panels A, C, E, G), 62.5 to 2000 μM for (α1,3-man)_2_, (Panels B, F) and 250 to 1000 μM for GlcNAcß1,2Man (Panels D, H). The apparent K_D _was calculated by steady-state affinity analysis. The curves represent a representative example of two independent experiments. The biosensor chip density was 7230 RU for GNA_maize _(or 120 fmol GNA_maize_) and 2455 RU for GNA (or 49 fmol GNA).

To verify the nature of the sugar specificity of GNA_maize _and GNA for gp120 binding, different glycan structures were evaluated for their binding capacity to immobilized GNA_maize _and GNA (Figure 4). Serial two-fold dilutions of (α1,2-man)_3 _[7.8-1000 μM], (α1,3-man)_2 _[62.5-2000 μM], (β1,4-GlcNAc)_3 _[7.8-1000 μM] and GlcNAcß1,2Man [250-1000 μM] were injected as analyte over immobilized GNA_maize _and GNA. The apparent K_D _was calculated by steady-state affinity analysis (Table 6). Under these experimental conditions, only GlcNAcß1,2Man was able to measurably bind to GNA_maize _but at rather low amplitudes. However, this oligosaccharide didn't bind to immobilized GNA. In contrast, (α1,2-man)_3 _and (α1,3-man)_2 _efficiently interacted with GNA at apparent affinity values (K_D_) of 1.50 mM and 4.44 mM, respectively, but did not bind to GNA_maize_. These findings confirm the striking glycan specificity shift of GNA_maize _when compared to GNA.

**Table 6:** Affinity data for the interactions of various oligosaccharides with immobilized GNA and GNA_maize_

Glycan	**K**_**D**_	
	
	GNA	**GNA**_**maize**_
(α1,2-man)_3_	1.5 ± 0.2 mM	N.D.^a^
(α1,3-man)_2_	4.4 ± 0.9 mM	N.D.
(ß1,4-GlcNAc)_3_	N.D.	N.D.
GlcNAcβ1,2Man	N.D.	binding detected^b^
GlcNAcβ1,2Manα1,3(GlcNAcβ1,2Manα1,6)Manβ1,4GlcNAcβ1,4GlcNAc	N.D.	binding detected^b^

^a^Not detectable. For these interactions no binding curves could be detected.

^b^Binding was observed but we were unable to determine the K_D_-value.

### Competition of GNA, GNA_maize _and mAb 2G12 for binding to HIV-1 gp120

**Figure 5:**
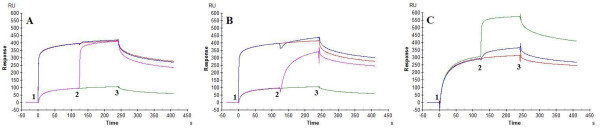
**Panel A: Competition experiment between GNA_maize _and GNA for binding to HIV-1 III_B _gp120 (chip density 400 RU ~ 3.3 fmol)**. 20 μM GNA_maize _were injected (time point 1, green and magenta), followed after 2 minutes by injection of 20 μM GNA_maize _(time point 2) in the absence (green) and presence of 5 μM GNA (magenta). Also 5 μM GNA (time point 1, red and blue) were injected followed by injection of 5 μM GNA (time point 2) in the absence (red) and presence of 20 μM GNA_maize _(blue). In Panel B, the competition experiment was performed between the plant lectins GNA and GNA_maize_, and the mAb 2G12 for binding to immobilized III_B _gp120. 20 μM GNA_maize _(green and magenta) and 5 μM GNA (red and blue) were injected as such (first 120 sec), followed by an additional injection of 3 μM 2G12 (next 120 sec) (in the continued presence of GNA_maize _[magenta] or GNA[blue]). In Panel C, 3 μM of 2G12 were injected (time point 1, red, blue and green) followed after 120 sec by an additional injection of 20 μM GNA_maize _(blue), 5 μM GNA (green) or by no injection (red) (time point 2) for another 120 seconds, in the continued presence of 3 μM 2G12.

To investigate whether GNA, GNA_maize _and 2G12 mAb compete for binding to immobilized gp120, the following experiment was performed (Figure 5). 20 μM GNA_maize _(green and magenta curves) or 5 μM GNA (red and blue curves) were administered for 2 minutes to gp120 immobilized on the sensor chip (Figure 5A, condition 1). Immediately at the end of the association phase (at 120 sec) 20 μM GNA_maize _was injected again as such (green curve) or in the presence of 5 μM GNA (magenta curve) for another 120 sec (Figure 5A, condition 2). After this time period, the dissociation phase was started (Figure 5A, condition 3). Likewise, in the GNA-binding experiment (red/blue curves), 5 μM GNA that was injected at condition 1, was injected after 120 sec again as such (red curve) or in the presence of 20 μM GNA_maize _(blue curve) for another 120 sec (Figure 5A, condition 2). Whereas the amplitude (RU) markedly further increased upon addition of 5 μM GNA to 20 μM GNA_maize _(~ 76% from the amplitude recorded when 5 μM GNA was injected as such), addition of 20 μM GNA_maize _to 5 μM GNA hardly further increased the amplitude afforded by GNA as such. These findings may indicate that GNA_maize _pre-binding to gp120 does not prevent additional GNA binding very much; however, GNA pre-binding seems to markedly preclude additional GNA_maize _binding. In panel B, a similar experiment was performed, but now it was the aim to evaluate whether the plant lectins compete with 2G12 for binding to immobilized gp120. In condition 1 of Figure 5B GNA_maize _(20 μM) (green and magenta curves) and GNA (5 μM) (blue and red curves) were injected and sustained for 120 sec till at the start of condition 2 when additional 2G12 (3 μM) (competing with GNA_maize _or GNA for binding to gp120) has been administered to the analyte (magenta and blue curves). Control curves where the initial compound injection is sustained without additional injection of another compound are green (GNA_maize_) and red (GNA). The data revealed that 2G12 could efficiently (~ 90%) bind to gp120 that contained pre-bound GNA_maize _(Figure 5B, magenta curve, condition 2) but not very efficiently (~ 20%) bind to gp120 that contained pre-bound GNA (Figure 5B, blue curve, condition 2). In panel C, 3 μM 2G12 was injected for 120 seconds (red curve) (condition 1). This concentration of 2G12 was kept in condition 2 of Figure 5C, but at that time point also 5 μM GNA (green curve), 20 μM GNA_maize _(blue curve) or no additional injection were administered (red curve). It was found that when 3 μM 2G12 were bound to gp120, ~ 70% of 5 μM GNA or ~ 85% of 20 μM GNA_maize _can still bind to gp120.

### Competition between PHA-E or SNA and GNA, GNA_maize _or mAb 2G12 for binding to HIV-1 gp120

**Figure 6:**
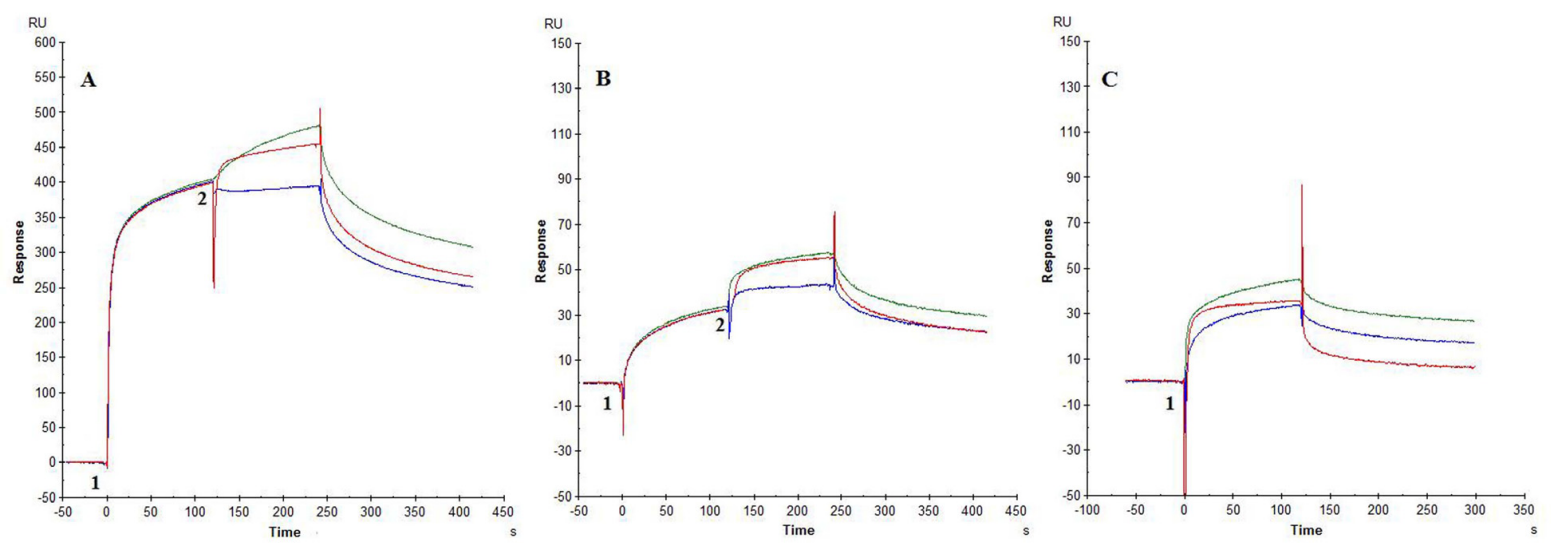
**Competition experiment between PHA-E (Panel A) or SNA (Panel B) with GNA_maize _(blue), GNA (green) or mAb 2G12 (red) for binding to HIV-1 gp120**. In Panel A, 2.5 μM of PHA-E were injected at time point 1, this concentration of PHA-E was sustained at time point 2 but now also 15 μM GNA_maize _(blue), 2.5 μM 2G12 (red) or 0.25 μM GNA (green) were additionally injected. A similar experiment was performed for 2.5 μM SNA (B). Control injections of 15 μM GNA_maize _(blue), 0.25 μM GNA (green) and 2.5 μM mAb 2G12 (red) are plotted in panel C.

A similar competition experiment was performed as described above, but 2.5 μM PHA-E (Figure 6A) or 2.5 μM SNA (Figure 6B) were injected at time point 1 and sustained at time point 2 at which additionally 15 μM GNA_maize _(blue), 2.5 μM 2G12 (red) or 0.25 μM GNA (green) were injected. The lectin PHA-E is known to preferentially bind to complex-type N-glycans through the recognition of Galβ1,4GlcNAc- and GlcNAcβ1,2Man-determinants [29]. SNA binds preferentially to sialic acid attached to galactose in α2,6- and to a lesser extent α2,3-linkage [30]. The data revealed that 0.25 μM GNA (green) and 2.5 μM 2G12 (red) can independently bind on PHA-E pre-bound gp120, whereas GNA_maize _(blue) could not bind any more to PHA-E pre-bound gp120 (Figure 6A). Likewise, the mAb 2G12 (red) and GNA (green) could rather efficiently bind to SNA pre-bound gp120 in contrast to GNA_maize _that only could partially bind to SNA pre-bound gp120 (Figure 6B). Control injections of 15 μM GNA_maize _(blue), 0.25 μM GNA (green) and 2.5 μM mAb 2G12 (red) are shown in Figure 6C.

### Homology modeling of GNA_maize_

**Figure 7:**
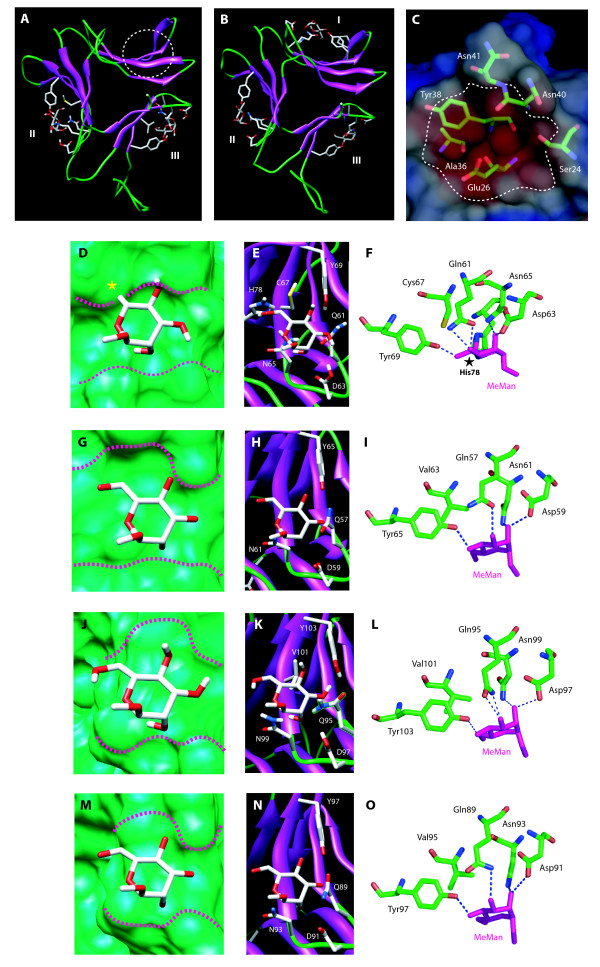
**Panel A and B: ribbon diagrams of GNA_maize _(A) and GNA (B) highlighting the mannose-binding sites I, II and III in both structures**. Panel C: electronegative cavity (white dotted line) in the region of site I of GNA_maize _(open white circle in Panel A) containing residues Ser24, Glu26, Ala26, Tyr38, Asn40 and Asn41 that could be involved in the binding of monosaccharides. Electronegative and electropositive potentials are colored red and blue, respectively. Neutral regions are colored white. Panel D,G,J and M: topography of site II of GNA_maize _(D) and GNA (G) and site III of GNA_maize _(J) and GNA (M) showing the anchoring of MeMan into the mannose-binding cavity. The yellow star indicates the protruding His78 residue that creates a steric clash with O6 of MeMan (D). The overall topography of the mannose-binding sites is indicated by red dotted lines. Panel E,H,K and N: ribbon diagrams showing the anchoring of MeMan into mannose-binding site II of GNA_maize _(E) and GNA (H) and site III of GNA_maize _(K) and GNA (N). Residues interacting with MeMan are in stick representation and are labelled. Panel F,I,L and O: stick representation of residues interacting with MeMan in site II of GNA_maize _(F) and GNA (I) and site III of GNA_maize _(L) and GNA (O). Hydrogen bonds are represented by deep blue dotted lines. Note the steric clash occurring between His78 and O6 of MeMan in site II of GNA_maize _(F).

Docking experiments performed with MeMan as a ligand suggested that GNA_maize _readily differs from GNA by the number of active carbohydrate-binding sites (Figure 7, Panels A and B). The GNA protomer possesses 3 active MeMan-binding sites which contain the conserved Gln-X-Asp-X-Asn-X-Val-X-Tyr monosaccharide-binding sequence (Figure 7, Panel B). Differences in the key residues that create a network of hydrogen bonds responsible for the binding of MeMan to site I of GNA rendered this binding site in GNA_maize _completely inactive. Except for a Val residue, which is replaced by a Cys residue in GNA_maize_, site II is apparently fully active; however the His78 of GNA_maize _(which replaces Ala in GNA) creates a steric clash with O6 of MeMan and prevents the monosaccharide to be correctly bound to the site (Figure 7, Panel D,E and F). Compared to site II of GNA (Figure 7, Panel G,H and I), site II of GNA_maize _should be devoid of any binding activity toward MeMan and Man. Finally, site III of GNA_maize_, which contains the unchanged key residues Gln95, Asp97, Asn99, Val101 and Tyr103 as in GNA, does not differ from site III of GNA (Figure 7, Panel M,N and O), and thus appears as the only active MeMan/Man-binding site in the GNA_maize _protomer (Figure 7, Panel J,K and L). These docking results fully support the reduced activity of GNA_maize _towards Man and high-mannose type glycans compared to GNA. In addition, the shape and size of the carbohydrate-binding cavities corresponding to sites II and III also differ between GNA_maize _and GNA (Figure 7, Panel D,G,J and M), which could account for the specificity of GNA_maize _towards complex glycans. Moreover, even though site I of GNA_maize _does not contain all the residues required for a proper binding of Man, this region possesses a deep electronegatively charged cavity (Figure 7, Panel C) that could serve as a monosaccharide-binding site for simple sugars different from Man, e.g. for GlcNAc.

## Discussion

Our antiviral data and previous observations [11] revealed that GNA and GNA_maize _both inhibit HIV-1 and HIV-2 infection. However GNA_maize _shows a strongly reduced anti-HIV-activity compared to GNA, being ~60- to ~100-fold less potent against HIV-1(III_B_) and HIV-2(ROD) infection. It was 30-fold inferior to inhibit giant cell formation between persistently HIV-1-infected HuT-78 cells and uninfected SupT1 cells, and it was 20- to 70-fold less efficient in inhibiting DC-SIGN-directed HIV-1 capture and subsequent transmission of DC-SIGN-captured HIV-1 particles to uninfected CD4^+ ^T-lymphocytes (Tables 1, 2, 3). The decreased antiviral activity is in agreement with the much lower affinity [~ 100-fold higher apparent affinity constant (K_D_)] that was recorded for the interaction between GNA_maize _and gp120 compared to GNA and gp120. This value points to a ~ 100-fold weaker binding of GNA_maize _than GNA to gp120. Thus, despite the high similarities at the sequence and structural level, both plant lectins have a strikingly different potency for their anti-HIV activity and interaction with their antiviral target (HIV gp120). Thus, the weaker contribution to the inhibitory effect against the HIV-1 infection by GNA_maize _is closely correlated with its weaker binding to HIV-1 gp120, presumably due to its carbohydrate specificity shift from oligomannose (for GNA) to complex-type glycans. In this respect, it cannot be excluded that the anti-HIV activity of GNA_maize _may be due, not only to a binding to complex-type glycans present on HIV-1 gp120 but also to potential binding to complex-type glycans of gangliosides that may be present in the virion envelope.

In the long-term drug selection experiments with GNA_maize_, one N-glycan deletion in gp120 (N301) was observed when all virus strains were taken into account (Table 4). The deletion represents a complex-type glycan deletion [28]. This *N*-linked sugar chain is the only one present in the V3-loop of the HIV-1 envelope. This complex-type *N*-glycan is conserved in most HIV-1 strains. The N301 glycan is in close proximity to important protein domains, in contrast to the complex glycans at V1/V2 or V4 of gp120. The V3 loop has been implicated in the binding of gp120 with CD4 and the chemokine secondary receptors [31]. It also plays a role in eliciting neutralizing anti-HIV antibodies [32,33]. Interestingly, the glycan present at N301 was earlier determined to be occupied by a tetraantennary complex glycan while most other complex type N-glycans are predominantly diantennary [34]. This finding may raise the possibility that a multivalent interaction with more than two antennae is favourable for GNA_maize _binding, although a glycan array revealed that GNA_maize _showed the highest binding affinities to biantennary (or monoantennary) GlcNAc β1-2Man-containing glycans [11]. In contrast, HIV-1 exposure to GNA resulted in the eventual deletion of 7 glycosylation sites of which 5 were high-mannose-type N-glycans (N230, N234, N289, N339 and N392) and only 2 complex-type N-glycans (N88 and N301) [35]. Similar preference for the deletion of high-mannose-type glycans has also been observed for the *Hippeastrum *hybrid (*Amaryllis*) lectin HHA [36], the prokaryotic lectin actinohivin [37,38], the cyanobacterial lectin Cyanovirin N [39], the 2G12 mAb [40] and the antibiotics pradimicin A and S [41,42]. Such unusual preference for deletion of high-mannose-type glycans is highly significant for these lectins since the glycan shield of the HIV-1 gp120 envelope, determined for gp120 expressed in Chinese hamster ovary (CHO) cells, exists of 11 high-mannose- or hybrid-type glycans and 13 complex-type glycans [28]. It was interesting to notice that one of the GNA_maize_-exposed virus strains also showed a glycosylation site deletion in gp41. It should, however, be kept in mind that the N811 position is located in the cytoplasmic tail of gp41 and thus was not supposed to be glycosylated in wild-type gp41. The relevance of the appearance of this mutation is therefore unclear. Also, the relevance of the formation of the new glycosylation motif at N29 in gp120 of one of the virus isolates is unclear because this amino acid is located in the membrane-embedded signal peptide and thus unlikely to be used for glycosylation.

Fouquaert and colleagues [11] demonstrated by glycan array analysis that GNA strongly interacts with high-mannose-type N-glycans and preferentially recognizes terminal mannose residues (Manα1,6Man > Manα1,3Man > Manα1,2Man), whereas GNA_maize _has poor, if any affinity for this type of glycans. In contrast, GNA_maize _recognizes complex N-glycans with a preference for a GlcNAc β1,2Manα1,3-X motif-containing glycan and/or a Neu5Acα2,6Galβ1,4-X motif-containing glycan. Thus, this surprising shift in glycan specificity from high-mannose-type to complex-type glycans between the closely related GNA and GNA_maize _explains the differences between both lectins in their preference for the nature (high mannose-type for GNA and complex-type for GNA_maize_) of the deletion of N-glycans in the drug resistance selection experiments. To further document this shift in sugar recognition we performed several surface plasmon resonance (SPR) experiments. In the first instance 5 oligosaccharides: (α1,2-man)_3_, (α1,3-man)_2_, (β1,4-GlcNAc)_3_, GlcNAcß1,2Man and GlcNAcβ1,2Manα1,3(GlcNAcβ1,2Manα1,6) Manβ1,4GlcNAcβ1,4GlcNAc were examined for binding to immobilized GNA and GNA_maize_. The SPR-results showed that only (α1,2-man)_3 _and (α1,3-man)_2 _preferentially bind to GNA but not GNA_maize _whereas GlcNAcß1,2Man and GlcNAcβ1,2Manα1,3(GlcNAcβ1,2Manα1,6) Manβ1,4GlcNAcβ1,4GlcNAc were able to bind to GNA_maize _but not to GNA. We found a slightly higher preference of GNA for (α1,2-man)_3 _than for (α1,3-man)_2 _whereas GNA was originally reported by Shibuya and co-workers [12] as a lectin with specificity towards oligosaccharides with terminal Manα1,3Man motifs. However, it should be noticed that in our SPR studies, a α1,3-man dimer but a α1,2-man trimer has been used. It is well known that often a higher degree of oligomerization results in a better affinity of the lectins for such sugar oligomers. The concomitant α1,2-man specificity of GNA is also in line with the glycan array data of Fouquaert *et al*. [11], and the α1,2-mannose oligomer affinity of GNA became also evident from the 2-fold lower K_D_-value of GNA binding to insect cell-derived gp120 (containing a high density of high-mannose-type glycan structures) than CHO cell-derived gp120 (Table 5). The 2-fold weaker affinity of GNA_maize _against insect cell-derived gp120 compared to CHO-derived HIV-1 gp120 is also in line with its predominant complex-type glycan specificity.

Epitope mapping experiments beween PHA-E (that prefers Galβ1,4GlcNAc- and GlcNAcβ1,2Man-linkages) or SNA (with Neu5Acα2,6Gal- and Neu5Acα2,3Gal-specificity) and GNA or GNA_maize _for binding to gp120 revealed that PHA-E pre-binding to gp120 prevents additional binding of GNA_maize_, in contrast to GNA, and SNA pre-binding of gp120 partially prevents the binding of GNA_maize _on gp120 but does not influence the additional binding of GNA to gp120. Taking into account the lectin-gp120 affinity data (Table 6) it can be concluded that the GNA_maize _lectin preferentially binds to GlcNAcβ1,2Manα1,3-X motifs and to a lesser, but still significant degree also to Neu5Acα2,6Galβ1-X motif determinants present on HIV-1 gp120. These data are in agreement with the findings of Fouquaert *et al*. [11] who demonstrated by glycan array analysis that GNA_maize _appears to prefer complex-type glycans containing GlcNAcβ1,2Man motifs and interactions with glycans containing Neu5Acα2,6Gal residues. When competition experiments between GNA, GNA_maize _and 2G12 for binding to gp120 were performed using SPR-analysis, GNA and GNA_maize _virtually bound independently of each other to gp120, although the amplitude of GNA decreased somewhat by 24% when gp120 was saturated with GNA_maize _(Table 7). Similar phenomena were observed with the α1,2-mannose specific anti-gp120 mAb 2G12 [43] binding of gp120: the binding signals of the snowdrop GNA lectin and the GNA_maize _lectin are diminished by 30% and 15% against 2G12 pre-bound gp120, respectively. These data prove that GNA has a more pronounced specificity for α1,2-man (competing for binding to the 2G12 epitope), in contrast to GNA_maize _which has rather weak, if any affinity (specificity) for α1,2-mannose oligomers.

**Table 7:** Competition of GNA, GNA_maize _and 2G12 mAb for binding to HIV-1 gp120

CBA	#RU at 2 min post injection	additional gp120 binding by the analyte (%)
5 μM GNA	409 ± 7	
20 μM GNA_maize_	111 ± 8	
3 μM 2G12	313 ± 48	
5 μM GNA + 20 μM GNA_maize_	38 ± 4	34 ± 1.4
20 μM GNA_maize _+ 5 μM GNA	310 ± 6	76 ± 0.2
3 μM 2G12 + 5 μM GNA	287 ± 5	70 ± 0.0
5 μM GNA + 3 μM 2G12	78 ± 5	25 ± 5.4
3 μM 2G12 + 20 μM GNA_maize_	93 ± 17	85 ± 21.3
20 μM GNA_maize _+ 3 μM 2G12	277 ± 4	89 ± 14.9

The Manα1,2-man oligomer-specific lectins [i.e. cyanovirin-N [39], Pradimicin A [41], Pradimicin S [42], actinohivin [38] and the mAb 2G12 [40]] and manα1,3/α1,6-man-oligomer specific lectins (i.e. GNA and HHA [8]) have previously been reported to contain potent anti-HIV activity. This manα1,2-, α1,3 or α1,6-man oligomer preference of GNA disappeared almost completely for the structurally closely related GNA_maize _and, likewise, resulted in a seriously decreased antiviral activity and a markedly lower affinity for HIV-1 gp120. These findings reveal the importance of interaction of CBAs with high-mannose-type glycans (preferentially manα1,2man) on the HIV gp120 envelope protein as a prerequisite to exhibit pronounced antiviral activity. Although the designation of complex *versus *high-mannose-type glycans on gp120 is based on the study of Leonard *et al*. [28] using monomeric recombinantly expressed gp120, it is well possible that the glycan content of the native gp120 trimer on the viral particles is somewhat different. In fact, Doores *et al*. [44] recently revealed that the envelope of native HIV virions, in sharp contrast to recombinantly gp120, almost exclusively contains an oligomannose (Man_5-9_GlcNAc_2_) glycan profile (< 2% complex-type glycans). However, it should be kept in mind that a proportion of the high-mannose-type glycans determined on virion trimeric gp120 can be derived from non-functional envelope forms of the virus containing a different glycosylation profile and therefore the amount of high-mannose-type glycans on the gp120 of virus particles can somewhat be overestimated in this study.

In conclusion, the markedly reduced effect in anti-HIV activity (up to ~100-fold) of GNA_maize _compared to GNA is explained by the shift in glycan recognition from high-mannose to complex-type glycans, and underscores the importance of efficient mannose-oligomer recognition of therapeutics as a prerequisite to exert significant anti-HIV activity. These findings would justify a rational design of new carbohydrate-binding therapeutics selectively targeting the high-mannose type glycans present on the HIV envelope gp120. Therefore, a better understanding of the molecular interaction between mannose-binding lectins such as actinohivin, cyanovirin, microvirin or griffithsin with α1,2-mannose oligomers by NMR or crystallography interaction studies would allow rational design of small synthetic carbohydrate (mannose)-binding agents. Also, (small-size) synthetic compounds such as borane-containing compound derivatives, known to specifically recognize configurations of two hydroxyl groups in *cis *(such as being present in mannose) [45,46] should be explored for gp120 binding and anti-HIV activity.

## Competing interests

The authors declare that they have no competing interests.

## Authors' contributions

BH participated in the design of the study, carried out cell cultures, SPR and virological experiments, and participated in manuscript writing. EJMVD supervised the production and isolation of the lectins. EF produced and purified the lectins. PR performed the modelling studies. KVL supervised and interpreted the sequence alignments. DS and JB designed and supervised the study, and participated in manuscript writing. All authors read and approved the final manuscript.
